# REAC Neurobiological Modulation With Neuro Postural Optimization (NPO) and Neuro Muscular Optimization (NMO) in Early Post-stroke Recovery: Functional Outcomes, Mechanistic Rationale, and Implications for Neurorehabilitation

**DOI:** 10.7759/cureus.91613

**Published:** 2025-09-04

**Authors:** Bruna Lombardi, Margherita Imbrenda, Valtere Giovannini, Vania Fontani, Salvatore Rinaldi

**Affiliations:** 1 Research, Rinaldi Fontani Foundation, Florence, ITA; 2 Physical Medicine and Rehabilitation, Azienda USL Toscana Centro, Prato, ITA; 3 Physical Medicine and Rehabilitation, Casa di Cura Villa Fiorita, Prato, ITA; 4 Regenerative Medicine, Rinaldi Fontani Institute, Florence, ITA

**Keywords:** early neurorehabilitation, functional dysmetria, functional recovery, neurobiological modulation, neuro muscular optimization, neuro postural optimization, non-invasive neuromodulation, post-stroke rehabilitation, reac technology

## Abstract

Stroke remains a leading cause of long-term disability worldwide, and early intervention is critical for optimizing neurorehabilitative outcomes by capitalizing on the heightened neuroplasticity of the acute and subacute phases.

This study aimed to evaluate whether the integration of Radio Electric Asymmetric Conveyer (REAC) neurobiological modulation protocols, Neuro Postural Optimization (NPO) and Neuro Muscular Optimization (NMO), into early post-stroke rehabilitation can accelerate and enhance functional recovery compared to conventional rehabilitation alone.

Thirteen patients (nine males, four females; age range: 56-86 years; mean: 74) received a single NPO session, followed by an intensive cycle of 10 NMO sessions distributed over five to six consecutive days. Treatment efficacy was evaluated using the Barthel Index (BI), the items from the mobility section of the Fugl-Meyer Assessment (FMA) motor function scale, and the assessment of functional dysmetria (FD). The REAC-treated cohort demonstrated marked improvements, with BI increasing from a mean of 33 to 68 and the items from the mobility section of the FMA from 9.0 to 17.3. FD, present in all patients at baseline, resolved completely after the NPO session and remained absent. A historical control group (n=13; 8 males, 5 females; age range: 56-94 years; mean: 76), matched for age and clinical condition, underwent standard rehabilitation without REAC interventions. This group showed more modest gains (BI from 36 to 67; items from the mobility section of the FMA from 8.9 to 15.3) despite a longer average hospitalization duration (34.4 days vs. 30.8 days in the REAC group). No adverse effects or complications were reported in the REAC-treated patients. These findings highlight the potential of REAC NPO and NMO protocols to support and enhance functional recovery when applied in the critical early stages of stroke rehabilitation. Further prospective controlled trials are warranted to validate these results and explore the mechanistic underpinnings of REAC-mediated neuromodulation in post-stroke recovery.

## Introduction

Stroke is a major global health concern, ranking among the leading causes of long-term disability and mortality [1]. In the aftermath of a stroke, early and effective neurorehabilitation is essential to leverage the intrinsic plasticity of the nervous system during the acute and subacute phases [2]. This critical window offers the greatest potential for functional recovery by re-engaging neural networks and promoting the reorganization of sensorimotor pathways.

Despite advances in conventional stroke rehabilitation techniques, many patients experience incomplete recovery and prolonged functional limitations [3]. Traditional therapeutic approaches are often limited by their reliance on peripheral mechanisms and require extended treatment durations to induce significant changes in motor function and autonomy [4]. This underscores the need for complementary strategies that can act upstream, enhancing central control and neuroplasticity from the earliest stages of intervention.

Radio Electric Asymmetric Conveyer (REAC) technology represents a novel non-invasive neurobiological modulation method that has shown promise in various clinical contexts. The REAC protocols Neuro Postural Optimization (NPO) [5,6] and Neuro Muscular Optimization (NMO) [7,8] are specifically designed to act on dysfunctional adaptive processes through endogenous bioelectric modulation [9,10]. NPO targets neuro-psycho-motor asymmetries by promoting functional reorganization of motor circuits [5], while NMO is intended to progressively restore neuromuscular coordination by acting on heterolateral agonist-antagonist muscle pairs across a sequence of sessions [7,8].

Previous studies have demonstrated the utility of REAC NPO and NMO protocols in conditions such as post-polio syndrome [7,8], orthopedic rehabilitation, and neurodegenerative disorders [11,12]. These protocols have been associated with improvements in motor coordination, reduction of dysfunctional motor patterns, and rebalancing of bioelectrical activity in affected neural circuits [13].

Given these premises, the present study investigates the application of REAC NPO and NMO protocols in a cohort of patients undergoing early post-stroke rehabilitation. We hypothesize that the integration of these neurobiological modulatory treatments into conventional care may lead to accelerated and more robust functional recovery compared to standard rehabilitation alone. Functional outcomes were assessed using the Barthel Index [14], the items from the mobility section of the Fugl-Meyer Assessment (FMA) [15], and the resolution of functional dysmetria, a clinically observable sign of neuromotor asymmetry. A historical control group, matched for age and clinical presentation, was included to provide a comparative reference.

## Materials and methods

Study design and setting

This was a prospective observational study conducted at Casa di Cura Villa Fiorita, Prato, Italy, within a defined clinical-scientific collaboration program with the Rinaldi Fontani Institute in Florence, Italy. The aim was to evaluate the effects of the REAC NPO and NMO protocols when integrated into early post-stroke rehabilitation, compared to standard rehabilitation alone.

Ethics statement

The study was conducted in accordance with the ethical principles outlined in the Declaration of Helsinki. All procedures involved treatments routinely used in clinical practice, and no experimental interventions or invasive methods were employed. The protocol was reviewed and approved by the Internal Review Board of the Rinaldi Fontani Institute (Protocol Number: IRB-RFI-2025-01-16-1). Written informed consent was obtained from all patients or their legal representatives for both the treatments administered and the anonymous use of clinical data.

Participants

Patients were consecutively recruited from hospital admissions if they presented with a subacute stroke confirmed by neuroimaging, functional deficits requiring neurorehabilitation, and no previous REAC treatment. Thirteen patients (nine males and four females; mean age 74 years, range 56-86) met these criteria and were included in the REAC group.

Intervention protocol

All REAC treatments were administered using the CE-certified REAC BENE 110 medical device (ASMED, Scandicci, Italy), which delivers asymmetrically conveyed very low-intensity radioelectric fields through an Asymmetric Conveyor Probe (ACP). All treatment protocols are pre-set, operator-independent, and cannot be modified, ensuring reproducibility across patients. The NPO protocol consisted of a single session aimed at optimizing neuro-psycho-motor function, delivered by placing the ACP on a specific auricular point, with a duration of a few milliseconds. The NMO protocol consisted of ten sessions over five to six consecutive days, with three to four sessions per day and a minimum interval of one hour between sessions. Each NMO session lasted approximately eight minutes and targeted heterolateral agonist-antagonist muscle pairs in a predefined sequence. All treatments were administered by clinicians trained in REAC methodology, following the same standardized procedures.

Assessment of functional dysmetria

The presence of functional dysmetria (FD), defined as an adaptive dysfunction of cerebellar origin characterized by asymmetric activation of motor responses during coordinated movements, was assessed through a specific, reproducible clinical maneuver [6]. This maneuver involves observing asymmetrical limb movements during the transition from supine to sitting position, allowing clinicians to detect dysfunctional motor coordination before and after the NPO session.

Outcome measures

Functional outcomes were assessed at admission (baseline) and discharge by physiatrists or physical therapists not involved in the delivery of REAC treatments, ensuring assessor blinding. The Barthel Index (BI) [14] was used to evaluate autonomy in activities of daily living. Motor impairment was assessed using only the items from the mobility section of the Fugl-Meyer Assessment (FMA) [15], with a scoring range from 0 (minimum function) to 27 (maximum function).

Comparison with the control group

For comparative purposes, data were analyzed alongside a historical control group of 13 patients (eight males and five females; mean age 76 years, range 56-94), matched for age, sex distribution, stroke subtype, baseline BI and FMA mobility scores, and rehabilitation setting. The control group underwent the same standard rehabilitation program as the REAC group, with the sole difference being the absence of REAC treatment.

Statistical analysis

Data were analyzed using descriptive statistics. Within-group comparisons were performed using paired t-tests or Wilcoxon signed-rank tests, and between-group comparisons using independent t-tests or Mann-Whitney U tests, as appropriate. Statistical significance was set at p < 0.05. Baseline comparability between groups was verified before analysis.

## Results

The REAC-treated group showed a significant improvement in both the Barthel Index and MI-27 scale from admission to discharge. The mean BI score [14] increased from 33.0 (SD ±14.8) to 68.0 (SD ±22.6), and the items from the mobility section of the Fugl-Meyer Assessment score [15] increased from 9.0 (SD ±6.4) to 17.3 (SD ±7.1), both changes statistically significant (p < 0.001). Functional dysmetria, initially present in all patients, resolved completely in each case after the NPO session and remained absent throughout the NMO treatment cycle [6].

The historical control group also showed improvement, but of a lesser magnitude. BI scores increased from a mean of 36.0 (SD ±13.7) to 67.0 (SD ±19.8), and MI-27 scores increased from 8.9 (SD ±6.2) to 15.3 (SD ±6.9). The improvements in the REAC group were more pronounced despite a shorter hospitalization period.

**Figure 1:**
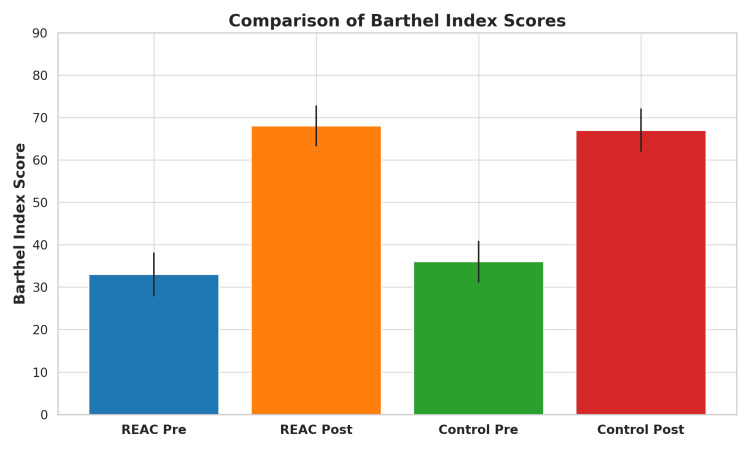
Comparison of Barthel Index scores before and after treatment in REAC-treated and control groups. Bar graph illustrating the mean ± standard deviation of Barthel Index scores at admission and discharge for both groups. The REAC-treated group (NPO + NMO) showed a greater mean improvement (from 33 to 68) compared to the historical control group undergoing conventional therapy (from 36 to 67). Although both groups improved significantly, the REAC group demonstrated a slightly higher gain with a shorter hospitalization period. This suggests a potential additive benefit of REAC neurobiological modulation in accelerating recovery of activities of daily living.

**Figure 2:**
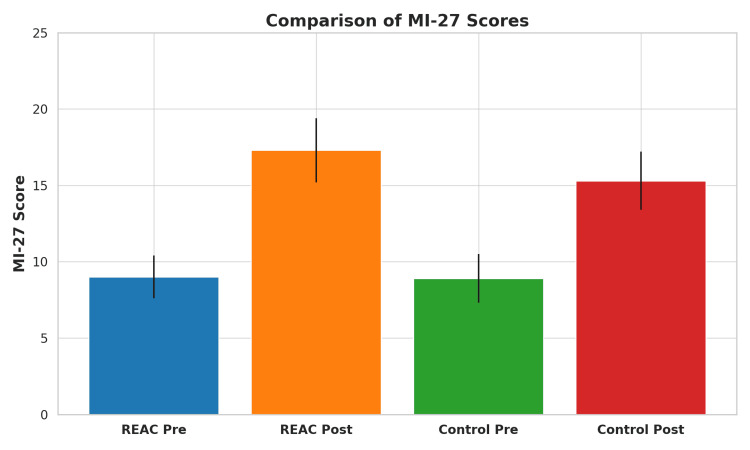
Comparison of MI-27 motor function scores before and after treatment in REAC-treated and control groups. Bar graph depicting the mean ± standard deviation of MI-27 scores at admission and discharge. The REAC-treated group exhibited a greater increase in motor performance (from 9.0 to 17.3) compared to the control group (from 8.9 to 15.3). The between-group difference in post-treatment MI-27 scores reached statistical significance (p = 0.041), supporting the potential central neuromotor benefits of REAC NPO and NMO protocols when integrated into early post-stroke rehabilitation.

Figure 1 and Figure 2 show bar graphs comparing the pre- and post-treatment mean values for both BI and MI-27 scores in REAC-treated versus control patients.

These visual representations highlight the greater magnitude of change in the REAC group, emphasizing the potential contribution of neurobiological modulation to functional recovery.

No adverse events, complications, or treatment interruptions were reported during the course of REAC NPO and NMO interventions.

## Discussion

The results of this study offer promising insights into the clinical potential of REAC neurobiological modulation when integrated into early post-stroke rehabilitation. The marked improvements observed in the REAC-treated group, particularly in the Barthel Index [14] and the mobility section items of the FMA scores [15], suggest that the combined use of the NPO and NMO protocols may enhance functional recovery by addressing both central and peripheral mechanisms of motor control [5,7,8,12].

In particular, compared to the historical control group, REAC-treated patients achieved superior functional gains within a shorter hospitalization period, despite similar initial levels of impairment, the only difference being the addition of REAC treatments. These differences underscore the potential of REAC neurobiological modulation as an upstream intervention capable of accelerating recovery trajectories in the early post-stroke phase.

The complete resolution of functional dysmetria following a single session of the NPO protocol in all treated patients stands out as a notable finding [6]. This outcome supports the hypothesis that NPO promotes rapid neuromotor reorganization involving cortical, subcortical, and cerebellar circuits [5] structures that are pivotal in regulating balance, inter-limb symmetry, and movement precision. Their modulation through NPO could therefore facilitate more effective postural and motor recovery.

The subsequent administration of the NMO protocol, delivered across multiple daily sessions targeting heterolateral agonist-antagonist muscle groups, appears to consolidate and extend the effects of NPO, with progressive and centrally mediated recruitment of neuromotor circuits that aligns with the broader reprogramming of dysfunctional motor patterns and may help to reinforce new coordination strategies [7,8,12,13].

These findings are consistent with the broader neurorehabilitation literature emphasizing the importance of early, neuroplasticity-oriented interventions in the subacute post-stroke window [16,17]. Reviews and meta-analyses indicate that interventions engaging central motor networks during this period yield the greatest functional gains [4], particularly on measures like the BI and FMA. The magnitude of improvement in our REAC-treated cohort compares favorably with outcomes typically reported for conventional therapy alone over similar or longer inpatient stays, suggesting a possible additive effect of upstream neuromodulation.

Evidence specific to REAC neuromodulation supports the plausibility of these effects [5,6]. A randomized, double-blind fMRI study demonstrated enduring changes in brain activation after a single REAC pulse, indicating rapid modulation of cortical networks relevant for motor control [5]. This rapid central effect is consistent with our immediate and stable resolution of functional dysmetria after NPO, an outcome previously linked to rebalancing cortico-subcortical-cerebellar dynamics [6]. Clinical studies in other neurological conditions, such as post-polio syndrome [7,8], Parkinson’s disease [11], and advanced Alzheimer’s disease [12], have also reported motor improvements difficult to explain by peripheral mechanisms alone, reinforcing the likelihood of a central, network-level mechanism relevant to post-stroke recovery.

It is particularly significant that patients with the lowest baseline BI and MI-27 scores demonstrated some of the most substantial improvements. This suggests that REAC neurobiological modulation may offer valuable benefits for patients with severe impairments, who typically face the greatest challenges in regaining autonomy.

The mechanisms underlying these effects are thought to involve the recovery of altered endogenous bioelectric activity [9,10], with downstream consequences including epigenetic modulation [18] and the functional reorganization of neural circuits [19]. Importantly, REAC protocols act only where dysregulations exist, without imposing external excitation, thereby preserving the system’s intrinsic capacity for self-correction.

This study has several methodological strengths. All REAC protocols are pre-set and operator-independent, ensuring reproducibility, and were delivered by clinicians trained in REAC methodology using standardized procedures. Functional assessments were performed by blinded evaluators who were not involved in treatment administration, thereby reducing observer bias. These aspects, already present in the original study design, have been made more explicit in the revised manuscript in response to reviewer comments, to facilitate transparency and replicability.

When compared with conventional physical modalities summarized in systematic reviews [2,20], our integrated NPO+NMO approach achieved comparable or greater gains despite a shorter mean length of stay. This pattern is in line with contemporary neurotechnology frameworks that advocate for therapies capable of modulating endogenous bioelectrical activity [21] and reducing maladaptive motor synergies, thereby accelerating learning during rehabilitation. Unlike other neuromodulation techniques that require precise cortical targeting or prolonged dosing to achieve clinical significance [22,23], the REAC protocols used here are brief, well-tolerated, and easily integrated into standard care without disrupting core rehabilitative practices [7,8,12].

Nevertheless, some limitations should be acknowledged. Our observational design and the use of a historical control group preclude definitive causal inference, and our outcome assessment focused on mobility-related FMA items without long-term follow-up. Furthermore, many comparative neuromodulation studies employ randomized controlled designs with domain-specific motor outcomes and extended monitoring, which should be incorporated into future research. Further prospective, randomized trials are needed to confirm these findings, and evaluate cost-effectiveness. Given their simplicity, safety, and ease of integration, REAC neurobiological modulation protocols warrant serious consideration for inclusion in early post-stroke rehabilitation strategies.

## Conclusions

This study demonstrates the potential role of REAC neurobiological modulation through the NPO and NMO protocols in enhancing early post-stroke functional recovery. The observed improvements in motor performance and autonomy, together with the rapid and stable resolution of functional dysmetria, suggest that REAC treatments may act upstream by facilitating neurofunctional reorganization and optimizing motor control circuits. The comparative analysis with a matched historical control group reinforces the clinical relevance of these findings, particularly considering the superior recovery achieved within a shorter rehabilitation timeframe. These results support REAC neuromodulation protocols as adjunctive tools in comprehensive post-stroke rehabilitation pathways. Future randomized controlled trials are warranted to further elucidate the long-term effects, cost-benefit ratio, and optimal integration of these innovative interventions into standard neurorehabilitation practices.

## References

[1] Feigin VL, Brainin M, Norrving B (2025). World Stroke Organization: Global Stroke Fact Sheet 2025. Int J Stroke.

[2] Li X, He Y, Wang D, Rezaei MJ (2024). Stroke rehabilitation: from diagnosis to therapy. Front Neurol.

[3] Roesner K, Scheffler B, Kaehler M, Schmidt-Maciejewski B, Boettger T, Saal S (2024). Effects of physical therapy modalities for motor function, functional recovery, and post-stroke complications in patients with severe stroke: a systematic review update. Syst Rev.

[4] Yan W, Lin Y, Chen YF, Wang Y, Wang J, Zhang M (2025). Enhancing neuroplasticity for post-stroke motor recovery: mechanisms, models, and neurotechnology. IEEE Trans Neural Syst Rehabil Eng.

[5] Rinaldi S, Mura M, Castagna A, Fontani V (2014). Long-lasting changes in brain activation induced by a single REAC technology pulse in Wi-Fi bands. Randomized double-blind fMRI qualitative study. Sci Rep.

[6] Fontani V, Rinaldi A, Rinaldi C (2022). Long-lasting efficacy of radio electric asymmetric conveyer neuromodulation treatment on functional dysmetria, an adaptive motor behavior. Cureus.

[7] André Nogueira JA, Souza Bulle Oliveira A, Pereira Motta M (2024). Neurobiological modulation with REAC technology: enhancing pain, depression, anxiety, stress, and quality of life in post-polio syndrome subjects. Sci Rep.

[8] Pereira Motta M, Oliveira AS, André Nogueira JA (2024). Efficacy of REAC neurobiological optimization treatments in post-polio syndrome: a manual muscle testing evaluation. J Pers Med.

[9] Elio C, Fontani V, Rinaldi S, Gasbarro V (2020). REAC-induced endogenous bioelectric currents in the treatment of venous ulcers: a three-arm randomized controlled prospective study. Acta Dermatovenerol APA.

[10] Fontani V, Cruciani S, Santaniello S, Rinaldi S, Maioli M (2023). Impact of REAC regenerative endogenous bioelectrical cell reprogramming on MCF7 breast cancer cells. J Pers Med.

[11] Rinaldi C, Landre CB, Volpe MI (2023). Improving functional capacity and quality of life in Parkinson's disease patients through REAC neuromodulation treatments for mood and behavioral disorders. J Pers Med.

[12] Olazarán J, González B, López-Álvarez J (2013). Motor effects of REAC in advanced Alzheimer's disease: results from a pilot trial. J Alzheimers Dis.

[13] Fontani V, Rinaldi S, Castagna A, Margotti ML (2012). Noninvasive radioelectric asymmetric conveyor brain stimulation treatment improves balance in individuals over 65 suffering from neurological diseases: pilot study. Ther Clin Risk Manag.

[14] Babov K, Plakida A, Balashova I, Zabolotna I, Gushcha S, Dmytro B (2025). Comparative assessment of Barthel index and functional independence measure in providing rehabilitation care for military personnel with combined injuries. Phys Rehabil Recreational Health Technol.

[15] Cecchi F, Carrabba C, Bertolucci F (2021). Transcultural translation and validation of Fugl-Meyer assessment to Italian. Disabil Rehabil.

[16] Aderinto N, AbdulBasit MO, Olatunji G, Adejumo T (2023). Exploring the transformative influence of neuroplasticity on stroke rehabilitation: a narrative review of current evidence. Ann Med Surg (Lond).

[17] Schevenels K, Gerrits R, Lemmens R, De Smedt B, Zink I, Vandermosten M (2022). Early white matter connectivity and plasticity in post stroke aphasia recovery. Neuroimage Clin.

[18] Levin M (2014). Endogenous bioelectrical networks store non-genetic patterning information during development and regeneration. J Physiol.

[19] Levin M (2021). Bioelectric signaling: reprogrammable circuits underlying embryogenesis, regeneration, and cancer. Cell.

[20] Filipska-Blejder K, Jaracz K, Ślusarz R (2025). Efficacy and safety of early mobilization and factors associated with rehabilitation after stroke-review. J Clin Med.

[21] Tang Y, Feng S, Yao K, Cheung SW, Wang K, Zhou X, Xiang L (2025). Exogenous electron generation techniques for biomedical applications: bridging fundamentals and clinical practice. Biomaterials.

[22] Bao SC, Khan A, Song R, Kai-Yu Tong R (2020). Rewiring the lesioned brain: electrical stimulation for post-stroke motor restoration. J Stroke.

[23] Qi S, Tian M, Rao Y, Sun C, Li X, Qiao J, Huang ZG (2023). Applying transcranial magnetic stimulation to rehabilitation of poststroke lower extremity function and an improvement: individual-target TMS. Wiley Interdiscip Rev Cogn Sci.

